# Potentially functional genetic variants in ferroptosis‐related *CREB3* and *GALNT14* genes predict survival of hepatitis B virus‐related hepatocellular carcinoma

**DOI:** 10.1002/cam4.6848

**Published:** 2023-12-27

**Authors:** Shicheng Zhan, Moqin Qiu, Xueyan Wei, Junjie Wei, Liming Qin, Binbin Jiang, Qiuping Wen, Peiqin Chen, Qiuling Lin, Xiaoxia Wei, Zihan Zhou, Yanji Jiang, Xiumei Liang, Runwei Li, Yingchun Liu, Hongping Yu

**Affiliations:** ^1^ Department of Experimental Research Guangxi Medical University Cancer Hospital Nanning China; ^2^ Department of Epidemiology and Health Statistics, School of Public Health Guangxi Medical University Nanning China; ^3^ Department of Respiratory Oncology Guangxi Medical University Cancer Hospital Nanning China; ^4^ Editorial Department of Chinese Journal of Oncology Prevention and Treatment Guangxi Medical University Cancer Hospital Nanning China; ^5^ Department of Clinical Research Guangxi Medical University Cancer Hospital Nanning China; ^6^ Department of Cancer Prevention and Control Guangxi Medical University Cancer Hospital Nanning China; ^7^ Scientific Research Department Guangxi Medical University Cancer Hospital Nanning China; ^8^ Department of Disease Process Management Guangxi Medical University Cancer Hospital Nanning China; ^9^ Department of Civil Engineering, College of Engineering New Mexico State University Las Cruces New Mexico USA; ^10^ Key Cultivated Laboratory of Cancer Molecular Medicine of Guangxi Health Commission Guangxi Medical University Cancer Hospital Nanning China; ^11^ Key Laboratory of Early Prevention and Treatment for Regional High Frequency Tumor (Guangxi Medical University) Ministry of Education Nanning China

**Keywords:** ferroptosis, hepatocellular carcinoma, overall survival, single nucleotide polymorphism

## Abstract

**Background:**

Ferroptosis is a known crucial player in the development of cancers. However, the effect of single nucleotide polymorphisms (SNPs) in ferroptosis‐related genes on survival in hepatitis B virus (HBV)‐related hepatocellular carcinoma (HBV‐HCC) patients remains unknown.

**Methods:**

We used two‐stage multivariable Cox proportional hazards regression analyses to estimate the associations between 48,774 SNPs in 480 ferroptosis‐related genes and overall survival (OS) of 866 HBV‐HCC patients.

**Results:**

We identified that two potentially functional SNPs (*CREB3* rs10814274 C > T and *GALNT14* rs17010547 T > C) were significantly independently associated with the OS of HBV‐HCC patients (CT + TT verse CC, hazards ratio (HR) = 0.77, 95% confidence interval (CI) = 0.67–0.89, *p <* 0.001 for rs10814274 and TC + CC verse TT, HR = 0.66, 95% CI = 0.53–0.82, *p <* 0.001 for rs17010547, respectively). Additional joint assessment of protective genotypes of these two SNPs showed that patients with 1–2 protective genotypes had a significantly better OS compared with those carrying 0 protective genotypes (HR = 0.56, 95% CI = 0.45–0.70, *p <* 0.001). Moreover, the expression quantitative trait loci (eQTL) analysis revealed that the survival‐associated SNP rs10814274 T allele was significantly correlated with reduced *CREB3* transcript levels in both normal liver tissues and whole blood cells, while the *GALNT14* rs17010547 C allele had a significant correlation with increased *GALNT14* transcript levels in whole blood cells.

**Conclusion:**

These results suggest that genetic variants of *CREB3* and *GALNT14* may affect the survival of HBV‐HCC patients, likely via transcriptional regulation of respective genes. However, further studies are required to confirm these findings.

## 1 INTRODUCTION

Primary liver cancer is the third most common cancer‐related death worldwide and the second leading cause of cancer deaths in China.^1^, ^2^ Approximately 90% of liver cancer patients were diagnosed with a histological subtype of hepatocellular carcinoma (HCC).^3^ Hepatitis B virus (HBV) infection has been found to be a major risk factor for HCC and accounts for 84% of HCC cases in China.^4^, ^5^ Meanwhile, published data showed that 87.7% of HCC patients were seropositive for HBV in southern China.^6^ Among the treatments for HCC patients, surgery is generally recommended.^7^ However, the prognosis remains at a poor 5‐year survival rate of only 18%.^4^ Continuous efforts have been made to improve HCC prognosis, and a series of clinical parameters were identified as predictive prognostic factors. For example, serum alpha‐fetoprotein (AFP) levels were found to have a negative impact on HCC prognosis.^7^ In addition, several classification systems have been derived and applied for the treatment strategy‐making and survival prediction for HCC patients, such as the Barcelona Clinic Liver Cancer (BCLC) stage and the TNM system.^8^, ^9^ However, HCC, as a highly heterogeneous cancer, has distinct prognoses for patients who showed similar clinical characteristics and received the same treatments. Therefore, it is crucial to identify additional prognostic biomarkers that address individual variability and help to improve risk stratification and treatment decision‐making for HCC patients.

As one of the most common forms of genetic variation, single nucleotide polymorphisms (SNPs) may affect gene expression and functions and thus have a significant impact on risk and prognosis of cancer.^10^ Genome‐wide association studies (GWAS) have detected several SNPs in genes such as *KIF1B*, *CDK14*, *GLUL*, *TEDDM1*, *STAT4*, *GRIK1*, and the *HLA* complex, which were associated with risk of HBV‐related HCC (HBV‐HCC).^11^, ^12^, ^13^, ^14^, ^15^ In addition, a recent GWAS has demonstrated some associations between SNPs and prognostic outcomes in HCC patients. For example, Li et al.^16^ reported that six SNPs on chromosomes 6p21 and 8p12 were not only associated with risk of chronic hepatitis HBV infection, or HBV‐HCC, but also overall survival (OS) of HBV‐HCC patients. More recently, Wei et al.^17^ identified five SNPs that might serve as a reliable predictive biomarker for survival of HCC patients. However, the vast majority of functional SNPs that did not reach the stringent *p*‐value after multiple test corrections may be ignored. Therefore, in the post‐GWAS era, a hypothesis‐driven approach using a fewer SNPs of candidate genes in a prognosis‐related biological pathway has been applied to avoid the nuisance of multiple tests and contribute to a more effective identification of potentially functional SNPs.^18^

Ferroptosis, a unique form of cell programming death, was first coined by Dixon et al. in 2012.^19^ Unlike other forms of cell death, such as necroptosis and apoptosis, ferroptosis was characterized by iron‐dependent lethal accumulation of lipid peroxides.^20^ Recently, a growing body of evidence has shown that ferroptosis plays an important role in cancer development and progression.^21^ With regard to HCC, several studies with experiments using HCC cell lines have demonstrated that ferroptosis might serve as the principal mechanism underlying the anticancer effect of sorafenib, suggesting a promising strategy for treating HCC by inducing ferroptosis.^22^ Meanwhile, recent studies have also demonstrated that ferroptosis‐related genes might serve as prognostic biomarkers for HCC patients. For example, one study using a prognostic model with ferroptosis‐related genes showed a great performance in predicting prognosis of HCC patients, and they found differences in immune infiltration levels between high‐ and low‐risk groups.^23^ Another study showed that four ferroptosis‐related genes (i.e., *FANCD2*, *CS*, *CISD1*, and *SLC1A5*) were positively associated with the progression of HBV‐HCC, of which higher expression levels of *SLC1A5* were associated with tumor progression, immunosuppression, and poorer prognosis in patients with HBV‐HCC.^24^ However, the roles of SNPs in ferroptosis‐related genes in HBV‐HCC remain unknown. In the present study, we tested the hypothesis that potentially functional genetic variants in the ferroptosis‐related genes are associated with survival of HBV‐HCC patients in a two‐stage analysis.

## 2 MATERIALS AND METHODS

### 2.1 Studied populations

A total of 866 histopathologically confirmed HCC patients from Guangxi Medical University Cancer Hospital were recruited between July 2007 and December 2017. More details about the population have been described elsewhere.^25^, ^26^ In brief, all patients were HBV seropositive and had undergone hepatectomy. Demographic and clinical variables, including age, sex, smoking status, drinking status, AFP level, cirrhosis, embolus, and BCLC stage, were collected. Among all patients, those with BCLC stage B or C had undergone operation according to the Chinese guidelines for treatment of HCC.^27^ All patients were followed up every 3 months within the first 2 years after hepatectomy and every 6 months in the next year through telephone calls. Survival time was from the date of surgery to the date of death or last follow‐up in March 2020. Each patient signed a written informed consent. The present study was approved by the Institutional Review Board of Guangxi Medical University Cancer Hospital (Approval Number: LW2023121).

### 2.2 Genotyping, Gene, and SNP Selecting

The whole genomic DNA of each HCC patient was extracted by a blood DNA extraction kit (Concert, Xiamen, China). Genotyping was performed using Illumina Infinium Global Screening Assay (Shanghai, China), and the quality control of raw data was described in detail elsewhere.^25^ We selected ferroptosis‐related genes from the FerrDB database (http://www.zhounan.org/ferrdb/current/).^28^ After removal of duplicated genes and genes in the sex chromosome, a total of 480 genes were selected as the candidate genes. All SNPs in candidate genes and within their ±2 kb flanking regions were extracted using Plink (version 1.09) (http://pngu.mgh.harvard.edu/purcell/plink/)^29^ and then screened by the following criteria: (1) with a calling rate less than 95%; (2) with minor allele frequency <0.05; 3) with Hardy–Weinberg equilibrium *p <* 1 × 10^−6^.

### 2.3 Expression quantitative trait loci (eQTL) analysis and functional prediction

To explore potentially functional SNPs, the eQTL analysis was performed for 208 normal liver tissues and 670 blood cells from the Genotype‐Tissue Expression (GTEx) database (https://www.gtexportal.org/).^30^ In addition, bioinformatics functional prediction was performed by using online tools: RegulomeDB (https://www.regulomedb.org/),^31^ HaploReg (https://pubs.broadinstitute.org/mammals/haploreg/haploreg.php),^32^ SNPinfo (https://snpinfo.niehs.nih.gov/snpinfo/snpfunc.html)^33^ and UCSC genome browser (https://genome.ucsc.edu/)^34^ to predict potential functions of the identified SNPs and other SNPs in high linkage disequilibrium (LD) in the same genes. Furthermore, the Kaplan–Meier plotter database was used to visualize the associations between the mRNA expression of genes and overall survival of HCC patients (https://kmplot.com/analysis/).^35^

### 2.4 Differential gene expression analysis

By using data from the TNMplot database (https://tnmplot.com/),^36^ we analyzed the differential gene expression levels between HCC tissues and paired adjacent normal tissues. To verify the expression levels of genes, we also analyzed the RNA sequencing data using newly collected tumor tissues and paired adjacent normal tissues from additional 100 HCC patients who had undergone hepatectomy in Guangxi Medical University Cancer Hospital.

### 2.5 Statistical analysis

All HCC patients were randomly assigned to discovery and replication groups in a 1:1 ratio. Then, with adjustment for demographic and clinical variables, multivariable Cox proportional hazards regression analysis was performed to evaluate the associations between SNPs and overall survival of HCC patients in an additive genetic model. The cut‐off *p*‐value in survival analyses was set at 0.05. Instead of using the stringent false discovery rate (FDR) because many SNPs were in LD as the results of the imputation, a false‐positive report probability (FPRP) analysis was performed with a prior probability of 0.1 to detect a Hazard Ratio (HR) of 1.5 for an association with alleles of each SNP. Only SNPs with FPRP values less than 0.2 in both discovery and replication datasets were selected for further analysis. A stepwise multivariable Cox regression model with adjustment for the above‐mentioned variables was used to identify independent SNPs. The stratification analysis by demographic and clinical variables of HBV‐HCC patients was also conducted to assess possible interactions between those variables and selected genotypes. The Kaplan–Meier curve was used to depict the associations between genotypes of SNPs and survival of HCC patients, as well as the combination of favorable genotypes. All statistical analyses were performed by R software (3.1.3 and 4.0.3 versions). The R packages used included “survminer”, “survival”, “gap”, and “GenABLE”.^37^ The Manhattan plot was drawn using the Haploview software.^38^ The regional plot was plotted using LocusZoom.^39^

## 3 RESULTS

### 3.1 Associations between SNPs in ferroptosis‐related gene and survival of HBV‐HCC patients

**FIGURE 1:**
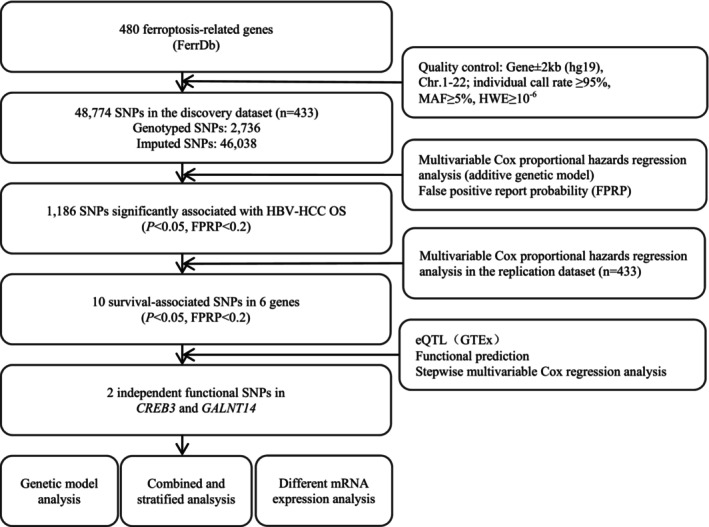
The overall procedures of the present study.

As shown in the flowchart of the study design (Figure 1), we randomly assigned the 866 patients into groups of discovery and replication. With detailed characteristics shown in Table S1, a total of 480 ferroptosis‐related genes were selected as the candidate genes. After quality control, 48,774 SNPs were available for further analysis in the discovery dataset. In the single‐locus analysis, 1186 SNPs were found to be associated with overall survival of HBV‐HCC patients (*p <* 0.05, FPRP<0.2), of which 10 SNPs remained significant after further assessment in the replication dataset (Table 1). The results of selected SNPs were visualized by Manhattan plots (Figure S1) and regional association plots (Figure S2). The baseline characteristics of 866 HBV‐HCC patients were described in Table S2.

**TABLE 1:** Associations between SNPs of ferroptosis‐related genes and survival of HBV‐HCC patients in discovery, replication, and combined dataset.

SNP	GENE	MAF	Discovery dataset (*n* = 433)				Replication dataset (*n* = 433)				Combined dataset (*n* = 866)			
			HR (95% CI)^a^	*p* ^a^	FDR	FPRP	HR (95% CI)^a^	*p* ^a^	FDR	FPRP	HR (95% CI)^a^	*p* ^a^	FDR	FPRP
rs10814274	*CREB3*	0.453	0.76 (0.62–0.94)	0.010	0.629	0.104	0.79 (0.65–0.96)	0.017	0.691	0.143	0.77 (0.67–0.89)	<0.001	0.611	0.004
rs6543592	*GALNT14*	0.177	0.73 (0.56–0.95)	0.019	0.701	0.187	0.66 (0.50–0.88)	0.004	0.572	0.081	0.69 (0.57–0.83)	<0.001	0.402	0.001
rs869199580	*GALNT14*	0.176	0.73 (0.56–0.95)	0.019	0.701	0.187	0.65 (0.49–0.87)	0.003	0.549	0.073	0.68 (0.56–0.83)	<0.001	0.402	0.002
rs17010547	*GALNT14*	0.176	0.73 (0.56–0.95)	0.019	0.701	0.187	0.65 (0.49–0.87)	0.003	0.549	0.073	0.68 (0.56–0.83)	<0.001	0.402	0.002
rs1214223169	*GALNT14*	0.310	0.71 (0.57–0.89)	0.003	0.483	0.036	0.77 (0.61–0.96)	0.020	0.691	0.168	0.73 (0.63–0.86)	<0.001	0.402	0.002
rs1399974412	*STMN1*	0.308	1.27 (1.04–1.56)	0.022	0.702	0.178	1.30 (1.06–1.60)	0.012	0.691	0.116	1.26 (1.09–1.46)	0.001	0.611	0.019
rs2900384	*GABARAPL1*	0.105	1.51 (1.11–2.05)	0.009	0.616	0.133	1.58 (1.17–2.13)	0.003	0.508	0.062	1.56 (1.26–1.93)	<0.001	0.402	0.001
rs7248	*GABARAPL1*	0.109	1.50 (1.10–2.04)	0.010	0.629	0.149	1.51 (1.13–2.04)	0.006	0.621	0.119	1.52 (1.23–1.88)	<0.001	0.402	0.002
rs8033106	*CPEB1*	0.202	1.35 (1.06–1.73)	0.015	0.684	0.167	1.32 (1.05–1.66)	0.017	0.691	0.155	1.32 (1.12–1.56)	<0.001	0.611	0.011
rs708563	*MAP3K14*	0.493	1.25 (1.03–1.52)	0.026	0.702	0.191	1.46 (1.19–1.78)	0.000	0.182	0.003	1.33 (1.15–1.53)	<0.001	0.402	0.001

Abbreviations: CI, confidence interval; FDR, false discovery rate; FPRP, false‐positive report probability; HR, hazards ratio; MAF, minor allele frequency; SNP, single nucleotide polymorphisms.

^a^ Adjusted for age, sex, smoking status, drinking status, cirrhosis, AFP, embolus, and BCLC stage.

### 3.2 The eQTL analysis and functional prediction

**FIGURE 2:**
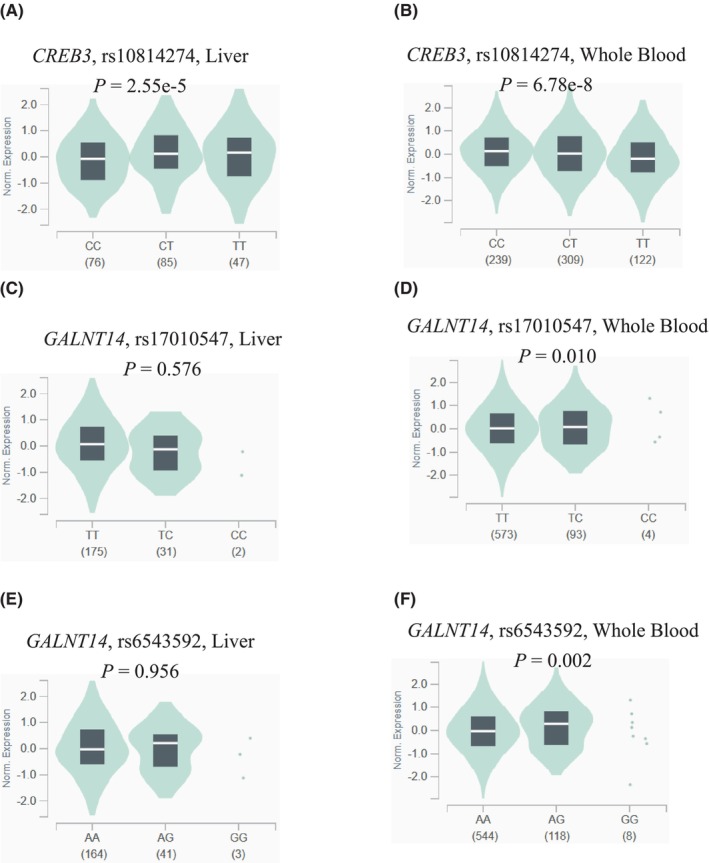
The expression quantitative trait loci (eQTL) analysis of three SNPs. The eQTL results of *CREB3* rs10814274 in normal liver tissues (A) and whole blood cells (B); the eQTL results of *GALNT14* rs17010547 in normal liver tissues (C) and whole blood cells (D); the eQTL results of *GALNT14* rs6543592 in normal liver tissues (E) and whole blood cells (F).

To identify those SNPs that may affect gene expression, we performed the expression quantitative trait loci (eQTL) analyses of 10 identified SNPs for their collations with mRNA expression levels of the corresponding genes by using data from the GTEx project. As shown in Figure 2, the *CREB3* rs10814274 T allele was correlated with decreased mRNA expression levels in both normal liver tissues and whole blood cells (NES = −0.15, *p <* 0.001; NES = −0.10, *p <* 0.001, respectively), compared with the C allele. Compared with the *GALNT14* rs17010547 T allele, the C allele was correlated with higher mRNA expression levels of gene in whole blood cells (NES = 0.098, *p =* 0.01) but not in normal liver tissues (NES = −0.058, *p =* 0.58). Meanwhile, the rs6543592 G allele was correlated with elevated mRNA expression levels of *GALNT14* in whole blood cells (NES = 0.1, *p =* 0.0023), compared with the A allele, but this trend was not significant in normal liver tissues (NES = 0.0052, *p =* 0.96). However, no significant correlation between the other seven SNPs and mRNA expression levels of their corresponding genes was found (Figure S3).

We then used online bioinformatics tools to further explore the functions of these three SNPs. According to the HaploReg, these three SNPs (*CREB3* rs10814274, *GALNT14* rs17010547, and *GALNT14* rs6543592) may disrupt the motif of several proteins, affecting the mRNA expression (Table S3). Moreover, *CREB3* rs10814274 was predicted to be located on the transcription factor binding site from SNPinfo (Table S3). This observation is supported by the experimental data from the ENCODE project, which suggest that *CREB3* rs10814274 is located in the region with enriched H3K4Me1 as well as the binding site of Rbpjl factor (Figure S4). On the other hand, *GALNT14* rs17010547 is likely to have an impact on the DNA enhancer (Table S3). Subsequently, the LD analysis showed that both rs17010547 and rs6543592 in *GALNT14* were in high LD (*r* ^2^ > 0.8; Figure S5). Having considered functional prediction and LD results, we selected *CREB3* rs10814274 and *GALNT14* rs17010547 for further analysis.

### 3.3 Identification of independent SNPs associated with HBV‐HCC survival

Next, a stepwise multivariable Cox regression analysis with adjustment for demographic and clinical variables was used to assess the independence of two selected SNPs. As a result, both *CREB3* rs10814274 C > T (HR = 0.76, 95% CI = 0.66–0.88 and *p <* 0.001) and *GALNT14* rs17010547 T > C (HR = 0.69, 95% CI = 0.58–0.83 and *p <* 0.001) remained statistically significantly associated with HBV‐HCC OS (Table S4). In addition, we performed 1000 times random validations with bootstrapping, and the results returned normally distributed HR values for both SNPs, suggesting rs10814274 and rs17010547 were not chosen by chance (Figure S6). Based on the above‐mentioned evidence, both *CREB3* rs10814274 and *GALNT14* rs17010547 were considered independently potentially functional SNPs and thus were selected for the final analysis.

### 3.4 Associations of *CREB3* rs10814274 and *GALNT14* rs6543592 genotypes with HBV‐HCC survival

**FIGURE 3:**
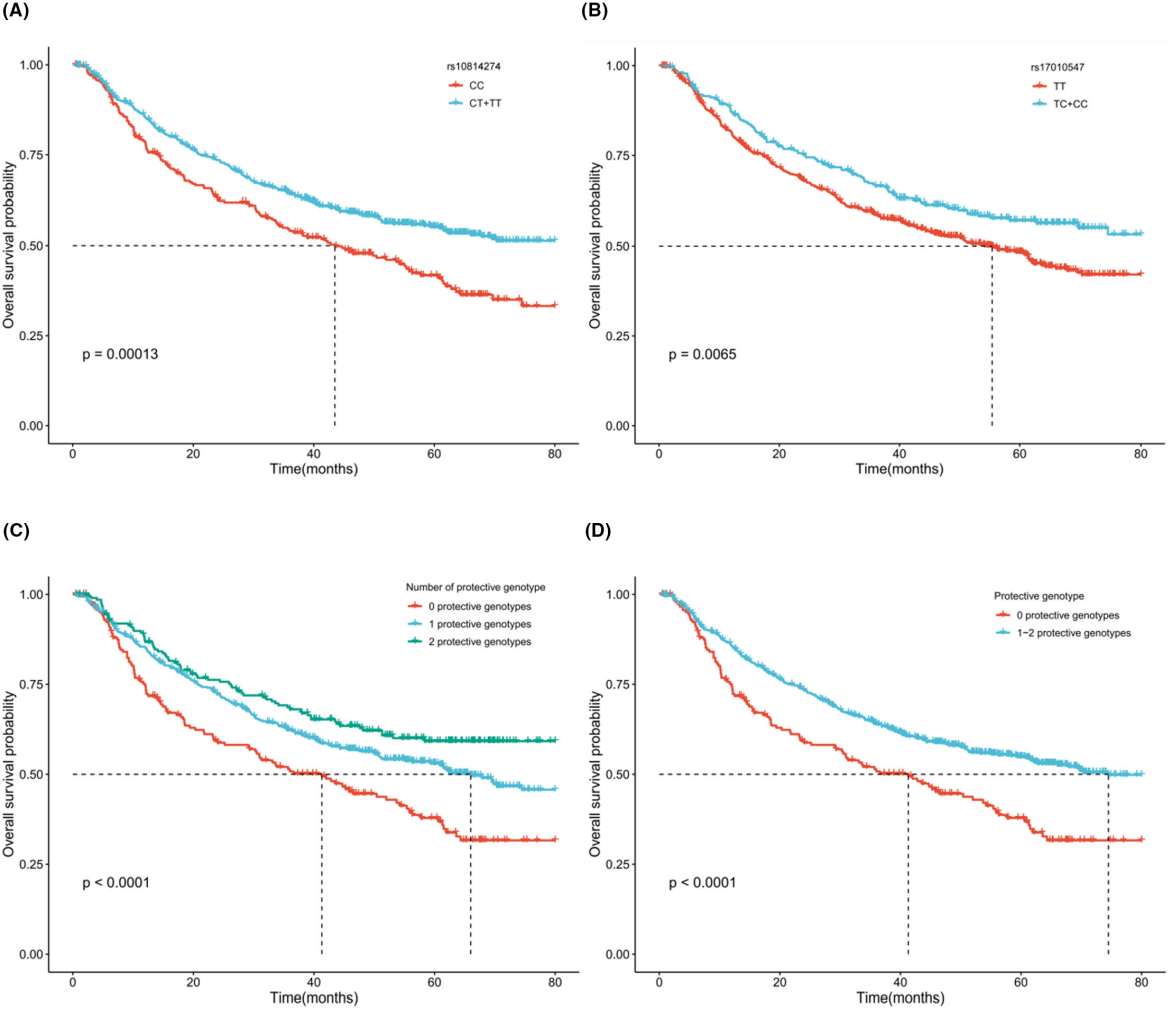
The Kaplan–Meier survival curves for 866 HBV‐HCC patients. Kaplan–Meier (KM) survival curves for HBV‐HCC patients by *CREB3* rs10814274 (A) and *GALNT14* rs17010547 (B) in dominant model. KM survival curves for HBV‐HCC patients by 0, 1, and 2 protective genotypes (C); by 0 and 1–2 protective genotypes (D).

When evaluating the associations of two selected SNPs with HBV‐HCC OS, we found that the HRs of both *CREB3* rs10814274 C > T and *GALNT14* rs17010547 T > C were larger than 1 (*p*_trend_ <0.001 for both SNPs). Specifically, patients with CT + TT genotypes of *CREB3* rs10814274 or TC + CC genotypes of *GALNT14* rs17010547 were associated with a better OS (HR = 0.62, 95% CI = 0.51–0.77, *p <* 0.001; HR = 0.66, 95% CI = 0.53–0.82, *p <* 0.001, respectively), compared with their wild genotypes. To assess the collective effect of two selected SNPs on HBV‐HCC OS, we combined their protective genotypes (i.e., *CREB3* rs10814274 CT + TT and *GALNT14* rs17010547 TC + CC) into a genetic score as the number of protective genotypes (NPG). We found that an increasing NPG was associated with a better OS in a dose‐dependent manner (*p*_trend_ <0.001). We also found that the risk of death for those patients with one or two NPG was about 44% lower (HR = 0.56) than for those who did not carry these protective genotypes (*p <* 0.001) (Table 2). These observed associations are visualized in the Kaplan–Meier survival curves (Figure 3).

**TABLE 2:** Associations between two selected SNPs and survival of HBV‐HCC patients.

Genotype	Frequency		Multivariable analysis	
	Number	Death (%)	HR (95% CI)^a^	*p* ^a^
*CREB3* rs10814274 C > T				
CC	261	150 (57.47)	1.00	
CT	425	186 (43.76)	0.62 (0.50–0.77)	<0.001
TT	180	83 (46.11)	0.64 (0.49–0.84)	0.001
Trend test				<0.001
CT + TT	605	269 (44.46)	0.62 (0.51–0.77)	<0.001
*GALNT14* rs17010547 T > C				
TT	592	304 (51.53)	1.00	
TC	244	105 (43.03)	0.68 (0.55–0.86)	<0.001
CC	30	10 (33.33)	0.45 (0.24–0.85)	0.015
Trend test				<0.001
TC + CC	274	115 (41.97)	0.66 (0.53–0.82)	<0.001
NPG^b^				
0	187	113 (60.43)	1.00	
1	479	228 (47.60)	0.62 (0.50–0.79)	<0.001
2	200	78 (39.00)	0.42 (0.31–0.56)	<0.001
Trend test				<0.001
0	187	113 (60.43)	1.00	
1–2	679	206 (30.34)	0.56 (0.45–0.70)	<0.001

Abbreviations: CI, confidence interval; HBV, hepatitis B virus; HCC, hepatocellular carcinoma; HR, hazards ratio; SNP, single nucleotide polymorphisms.

^a^ Adjusted for age, sex, smoking status, drinking status, cirrhosis, AFP, embolus, and BCLC stage.

^b^ NPG: number of protective genotypes (rs10814274 CT and TT; rs17010547 TC and CC).

### 3.5 Stratified analysis between protective genotypes and HBV‐HCC survival

To evaluate whether the combined effect of NPG on HBV‐HCC survival was modified by other variables, we performed stratified analysis by age, sex, smoking status, drinking status, cirrhosis, AFP, embolus, and BCLC stage. As shown in Table 3, those patients with one or two NPG had a better OS in all subgroups except for female patients. No interactive effects between the variables and survival‐associated genotypes were observed in all subgroups, except for an interaction between NPG and AFP (*p*_interaction_ = 0.024), which may be confounded by some unknown factors.

**TABLE 3:** Stratified analysis of the protective genotypes of selected SNPs in HBV‐HCC patients.

Characteristics	0 NPG^a^		1–2 NPG^a^		Multivariable analysis		*p*_inter_ ^c^
	All	Death (%)	All	Death (%)	HR (95% CI)^b^	*p* ^b^	
Age							0.172
≤47	105	57 (54.28)	327	129 (39.44)	0.63 (0.46–0.86)	0.004	
>47	82	56 (68.29)	352	177 (50.28)	0.49 (0.35–0.66)	<0.001	
Sex							0.658
Female	18	8 (44.44)	88	34 (38.63)	0.62 (0.27–1.42)	0.26	
Male	169	105 (62.13)	591	272 (46.02)	0.54 (0.43–0.69)	<0.001	
Smoking status							0.162
No	111	61 (54.95)	434	207 (45.93)	0.68 (0.51–0.92)	0.011	
Yes	76	52 (68.42)	245	99 (40.40)	0.41 (0.29–0.58)	<0.001	
Drinking status							0.149
No	122	66 (54.09)	492	226 (45.93)	0.64 (0.48–0.84)	0.002	
Yes	65	47 (72.30)	187	80 (42.78)	0.43 (0.30–0.62)	<0.001	
Cirrhosis							0.956
No	84	54 (64.28)	306	130 (42.48)	0.48 (0.34–0.66)	<0.001	
Yes	103	59 (57.28)	373	176 (47.18)	0.60 (0.45–0.82)	0.001	
AFP (ng/mL)							0.024
≤400	116	65 (56.03)	406	167 (41.13)	0.59 (0.44–0.79)	<0.001	
>400	71	48 (67.60)	273	139 (50.91)	0.51 (0.36–0.72)	<0.001	
BCLC stage							0.349
0/A	83	36 (43.37)	344	110 (31.97)	0.65 (0.44–0.97)	0.027	
B/C	104	77 (74.03)	335	196 (58.50)	0.52 (0.39–0.68)	<0.001	
Embolus							0.109
No	134	71 (52.98)	502	189 (37.64)	0.57 (0.43–0.75)	<0.001	
Yes	53	42 (79.24)	197	117 (59.39)	0.53 (0.37–0.77)	<0.001	

Abbreviations: CI, confidence interval; HBV, hepatitis B virus; HCC, hepatocellular carcinoma; HR, hazards ratio; SNP, single nucleotide polymorphisms.

^a^ NPG: number of protective genotypes (rs10814274 CT and TT; rs17010547 TC and CC).

^b^ Adjusted for age, sex, smoking status, drinking status, cirrhosis, AFP, embolus, and BCLC stage.

^c^ *p*_inter_: *p*‐value for interaction analysis between NPG and covariables.

### 3.6 Different mRNA expression analysis

**FIGURE 4:**
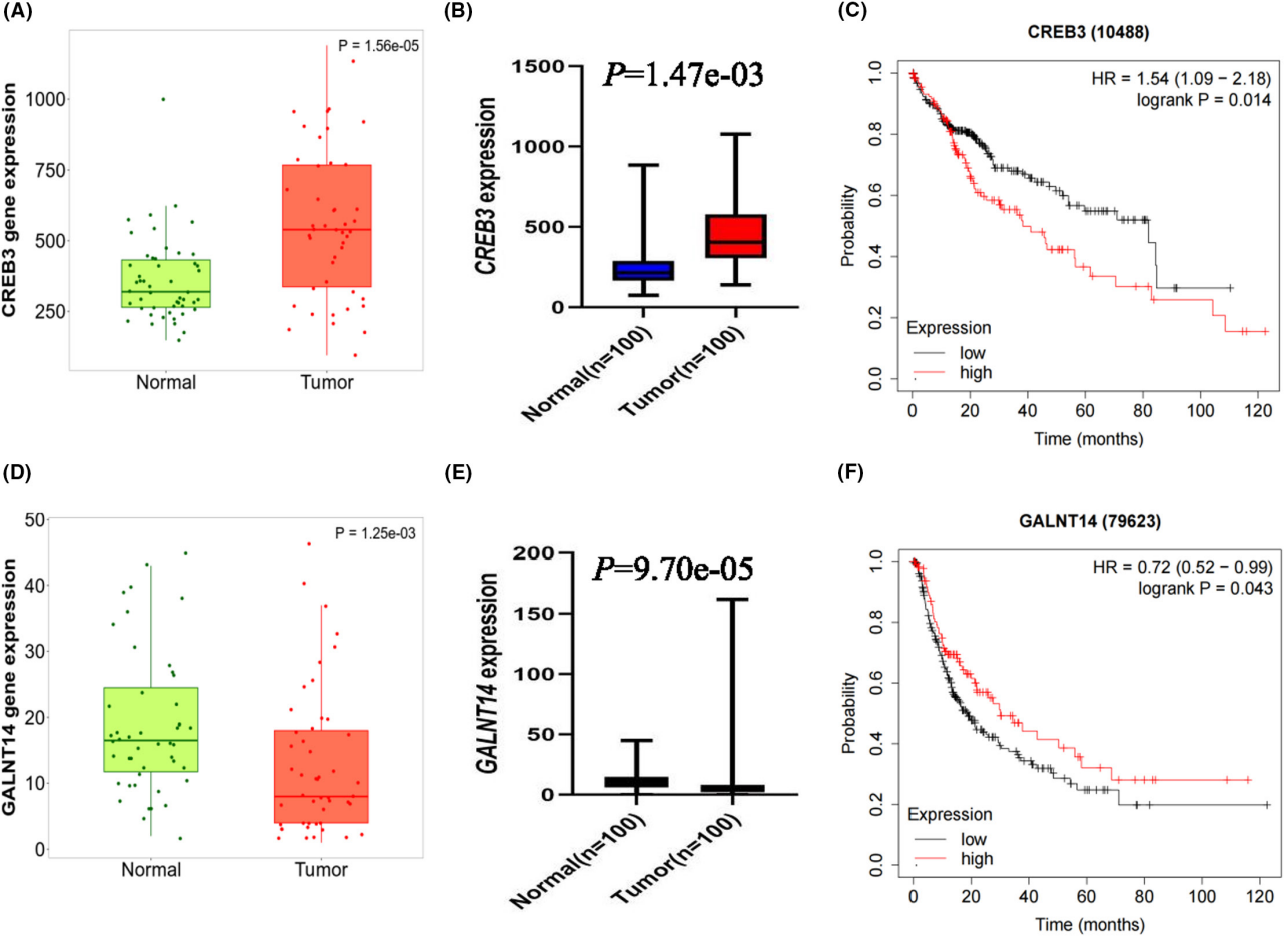
Differential mRNA expression analysis and overall survival analysis of the two genes. The expression level of *CREB3* mRNA from TNMplot (A) and our data (B). The KM survival curves for *CREB3* mRNA expression levels with OS (C) of HCC patients. The expression level of *GALNT14* mRNA from TNMplot (D) and our data (E). The KM survival curves for *GALNT14* mRNA expression levels with PFS of HCC patients (F). OS, overall survival; PFS, progression‐free survival.

To further explore the roles of *CREB3* and *GALNT14* in HCC survival, we assessed the mRNA expression levels of these two genes in paired HCC tumors and adjacent normal tissues, as well as the correlation between gene expression levels and survival of HCC patients. As shown in Figure 4, the expression levels of *CREB3* were higher in the tumor tissues, and higher expression levels were associated with a poorer HCC OS **(**Figure 4A–C **)**. Interestingly, the expression levels of *GALNT14* were lower in tumor tissues, and higher expression levels of *GALNT14* were associated with a better progression‐free survival (PFS) (Figure 4D–F), but the correlations between *GALNT14* expression levels and HCC OS were not significant.

## 4 DISCUSSION

In the present study, we evaluated the associations between 48,774 SNPs of 480 ferroptosis‐related genes and HBV‐HCC survival in multivariable Cox regression analysis. We demonstrated that both *CREB3* rs10814274 C > T and *GALNT14* rs17010547 T > C were associated with a better OS of HBV‐HCC patients. The underlying mechanism for the observed effects of both SNPs on HCC survival is likely through the regulation of their corresponding gene expression.

In ferroptosis, many genes participate in cancer‐associated signaling pathways, leading to cell death, which may affect tumor progression and response to treatment. However, few have studied the roles of ferroptosis‐related genes in tumor progression and clinical outcomes. For example, one study reported that genetic variants of ferroptosis‐related *APOE*, *BCL3*, and *ALOX5AP* were associated with the risk of thyroid cancer.^40^ Another study showed that SNPs in *IDH1* ^*105CCT*^ were associated with ferroptosis in inflammatory intrahepatic cholangiocarcinoma (ICC).^41^ To the best of our knowledge, the present study is the first to report associations between genetic variants in ferroptosis‐related genes and survival of HBV‐HCC patients.

*CREB3* belongs to the cyclic AMP‐response element‐binding protein (CREB) family. While *CREB3* functions as a transcription factor that regulates numerous genes in the central nervous system and responds to the Golgi stress,^42^, ^43^ the whole CREB family is considered often overexpressed and functions as a tumor mediator in multiple cancers.^44^ For liver cancer, previous studies showed that inhibition of CREB could decrease tumor cell proliferation, and higher expression of CREB was associated with a worse prognosis, indicating an oncogenic effect on liver cancer.^45^, ^46^, ^47^, ^48^ Similarly, Shen et al. reported that the mRNA expression levels of *CREB3* were higher in HCC tissues than in adjacent normal tissues, and higher expression of *CREB3* was associated with a worse 10‐year overall survival in patients with HCC.^49^ As for HBV infection, recent studies have demonstrated that CREB plays a crucial role in HBV replication. For example, one study demonstrated that a CREB motif in the HBV pre‐S2 region contributed to basal S promoter activity, and they also found that CREB/PKA signal transduction pathways in hepatocytes may be utilized by HBV to enhance HBsAg expression during homeostasis and hepatic inflammation.^50^ Another study showed that both replication and gene expression of HBV require functional CREB and HBV‐CRE, and CRE decoy oligonucleotides and the overexpression of CREB mutants could effectively block the HBV life cycle.^51^ Notably, it has been reported that ferroptosis was negatively regulated by CREB in lung adenocarcinoma. To be more specific, CREB could positively regulate the transcription level of glutathione peroxidase 4 (GPX4) through binding to its promoter region, which is marked by the upregulation of lipid reactive oxygen species (ROS). Knockdown of CREB led to ferroptotic‐like cell death, which also supports the oncogenesis role of CREB.^52^ Therefore, we speculate that changing expression levels of *CREB3* may alter the inhibiting effect of ferroptosis in HCC cells and subsequently affect the prognosis of HCC. In the present study, we found that the *CREB3* rs10814274 T allele was associated with a better survival of HBV‐HCC patients and a significant association of decreased *CREB3* mRNA expression levels in both liver tissues and whole blood cells. In addition, the *CREB3* mRNA expression levels were much higher in HCC tissues than in adjacent normal tissues, and higher expression levels of *CREB3* mRNA were associated with a worse prognosis in HCC patients, which also supports the oncogenesis role of *CREB3*. However, these findings need to be substantiated in further molecular biology experiments and clinical studies.

*GALNT14*, located on chromosome 2, is one of the polypeptide N‐acetylgalactosaminyltransferase (GALNT) family members. The GALNT family could catalyze protein O‐glycosylation, and the abnormal expression of GALNT could lead to distinct results through alteration of O‐glycosylation in several cancers.^53^, ^54^ Although the mechanisms of *GALNT14* in HCC are largely unclear, it was reported that *GALNT14* promoted cell proliferation and migration, and silencing *GALNT14* enhanced cell sensitivity to anticancer drugs, implying an important functional role of *GALNT14* in HCC.^55^ Concerning ferroptosis, Li et al.^56^ reported that downregulation of *GALNT14* could induce ferroptosis by inhibiting the mTOR/EGFR pathway, which then suppresses the protein levels of SLC7A11 and GPX4. This anti‐ferroptosis role is consistent with the growth‐promoting role of *GALNT14* in HCC, but the role of *GALNT14* in HCC has not yet been fully elucidated. In the present study, we found that lower expression of *GALNT14* was associated with a worse PFS, implying that *GALNT14* may play a tumor suppressor role in HCC, but the molecular mechanisms of *GALNT14* in predicting survival of HCC patients need to be further investigated. Moreover, we found that the *GALNT14* rs17010547 C allele was associated with a protective effect on HBV‐HCC survival and a significant association of increased *GALNT14* mRNA expression levels in whole blood cells, but the underlying mechanisms also need to be further investigated.

One previous GWAS study has demonstrated the association between the *GALNT14* rs9679162 genotypes and the response to chemotherapy in advanced HCC patients.^57^ In later studies, rs9679162 was also found to be an effective predictor in HCC patients who received transarterial chemoembolization (TACE) or sorafenib treatment.^58^, ^59^ However, by using data from 160 patients with advanced HCC, Chu et al.^60^ did not find any significant association between rs9679162 genotypes and survival of HCC patients. Consistent with their findings, we did not find any significant associations between rs9679162 genotypes and survival of HBV‐HCC patients in the present study (Table S5), and rs9679162 was not in LD with rs17010547. One possible explanation is the relatively small sample size in our study, which may limit the power to detect a weak association between rs9679162 and HBV‐HCC survival, particularly in HBV‐HCC patients who had undergone hepatectomy. Therefore, additional experimental investigations are required to determine how *GALNT14* rs17010547 T > C may influence HCC.

Several inherent limitations should be mentioned in the present study. Firstly, all patients were recruited in the same hospital, which might cause selection bias. Secondly, the sample size of the present study was relatively small, and further studies with a larger population are needed to verify our findings. Lastly, the molecular mechanisms underlying the survival‐associated SNPs and the observed association call for direct biological experiments for functional validation.

In summary, the present study identified statistically significant associations between two potentially functional genetic variants (*CREB3* rs10814274 and *GALNT14* rs17010547) in ferroptosis‐related genes and survival of HBV‐HCC patients. The protective genotypes of these two SNPs contributed to a better OS in a dose–response manner in the combined analysis. Such protective effects on OS are likely through SNP‐associated expression regulation of *CREB3* and *GALNT14*. These results provide some valuable information for risk stratification and treatment strategy‐making for HBV‐HCC patients.

## AUTHOR CONTRIBUTIONS

**Shicheng Zhan:** Data curation (lead); writing – original draft (lead). **Moqin Qiu:** Methodology (equal); writing – review and editing (lead). **Xueyan Wei:** Software (equal). **Junjie Wei:** Data curation (equal). **Liming Qin:** Data curation (equal). **Binbin Jiang:** Data curation (equal). **Qiuping Wen:** Methodology (equal). **Peiqin Chen:** Methodology (equal). **Qiuling Lin:** Methodology (equal). **Xiaoxia Wei:** Methodology (equal). **Zihan Zhou:** Software (equal). **Yanji Jiang:** Software (equal). **Xiumei Liang:** Investigation (lead). **Runwei Li:** Writing – review and editing (supporting). **Yingchun Liu:** Conceptualization (supporting); methodology (supporting); supervision (equal). **Hongping Yu:** Conceptualization (lead); methodology (lead); resources (lead).

## FUNDING INFORMATION

This study was supported by the Key Laboratory of Early Prevention and Treatment for Regional High Frequency Tumor, Ministry of Education (Grant No. GKE‐ZZ202104), the Natural Science Foundation of Guangxi Province (Grant No. 2023GXNSFBA026091 and 2023GXNSFBA026201), the Guangxi Medical and health appropriate Technology opening and popularization and application project (Grant No. S2022112), the Youth Science Foundation of Guangxi Medical University (Grant No. GXMUYSF 202312 and GXMUYSF 202304) and the Youth Program of Scientific Research Foundation of Guangxi Medical University Cancer Hospital (YQJ2022‐5).

## CONFLICT OF INTEREST STATEMENT

All authors declare no potential competing interests are disclosed.

## ETHICS STATEMENT

All patients recruited in the present study signed an informed consent, and this study was approved by Guangxi Medical University Cancer Hospital (LW2023121).

## Supporting information


Appendix S1.

## Data Availability

The data that support the findings of this study are available from the corresponding author upon reasonable request.
