# Supplementary material for: Potentially functional genetic variants in ferroptosis‐related CREB3 and GALNT14 genes predict survival of hepatitis B virus‐related hepatocellular carcinoma
Source: Cancer Med. 2023 Dec 27;13(1):e6848. doi: 10.1002/cam4.6848 (PMC10807646; doi:10.1002/cam4.6848)
Supplement: Supplementary file 1 — Appendix S1. [file CAM4-13-e6848-s001.docx]

| **A** |
| --- |
| 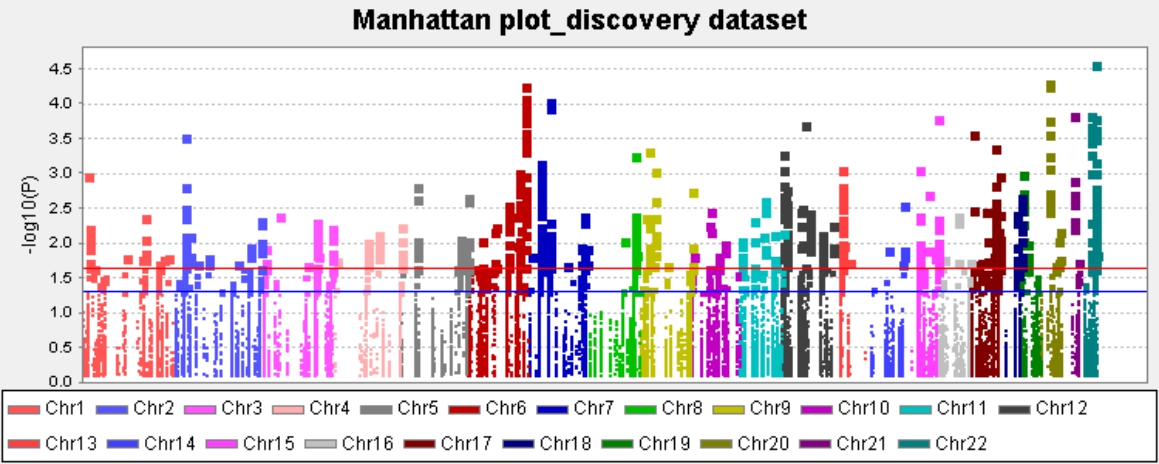 **FPRP = 0.2**  ***P* = 0.05** |
| **B** |
| 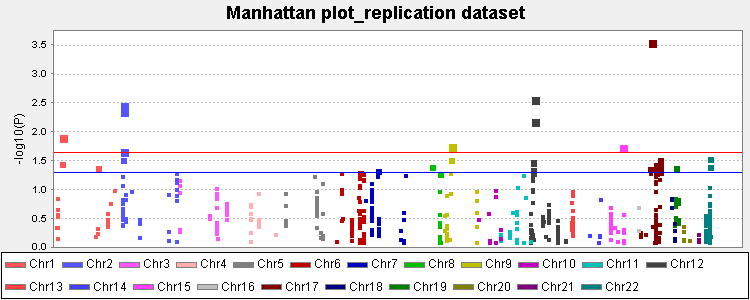 ***CREB3* rs10814274 C>T**  ***GALNT14* rs17010547 T>C**  **FPRP = 0.2**  ***P* = 0.05** |
| **Figure S1**. Manhattan plot. The Manhattan plot for 48,774 SNPs in the discovery dataset (A). The Manhattan plot for 1186 SNPs in the replication dataset (B). The blue horizontal line indicates *P*-value equal to 0.05, and the red horizontal line represents an FPRP value equal to 0.2. |

| **A** |
| --- |
| **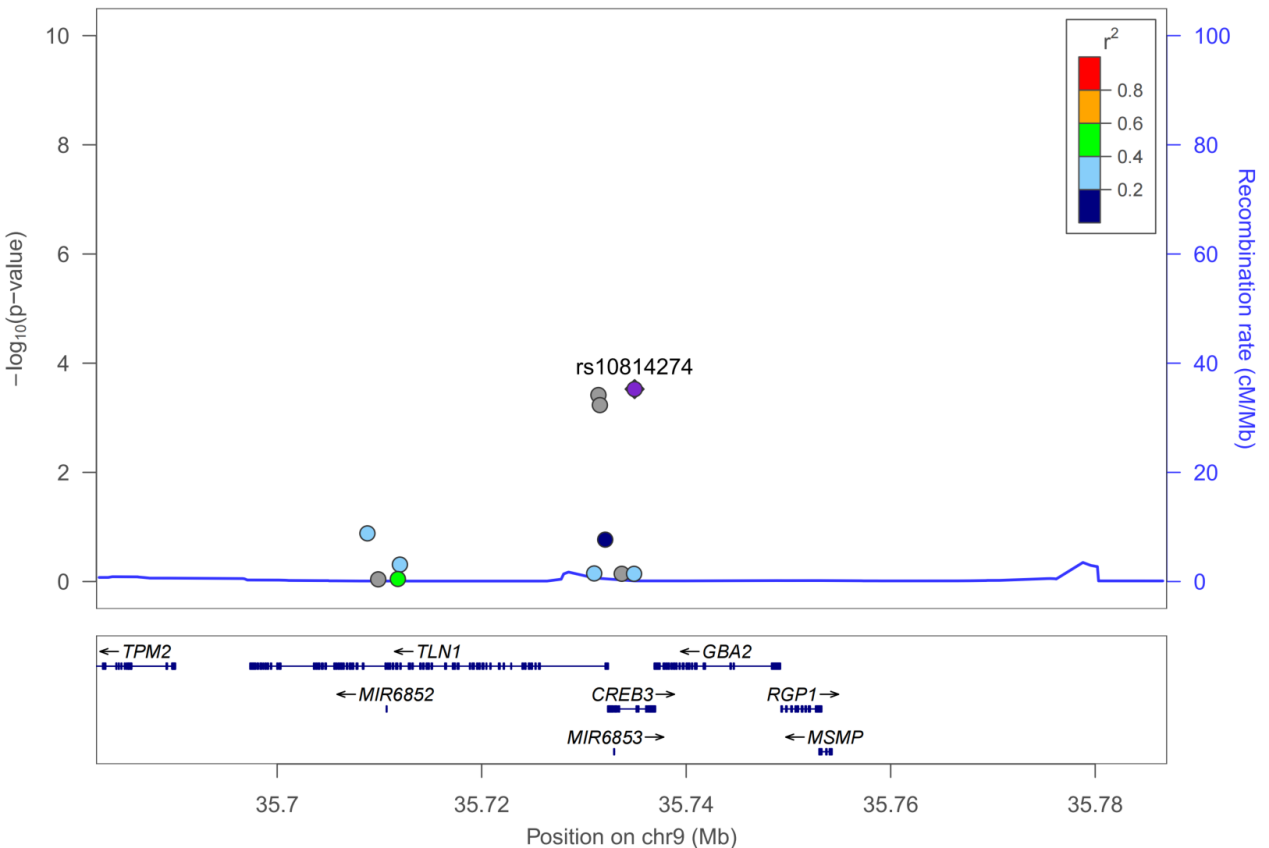** |
| **B** |
| 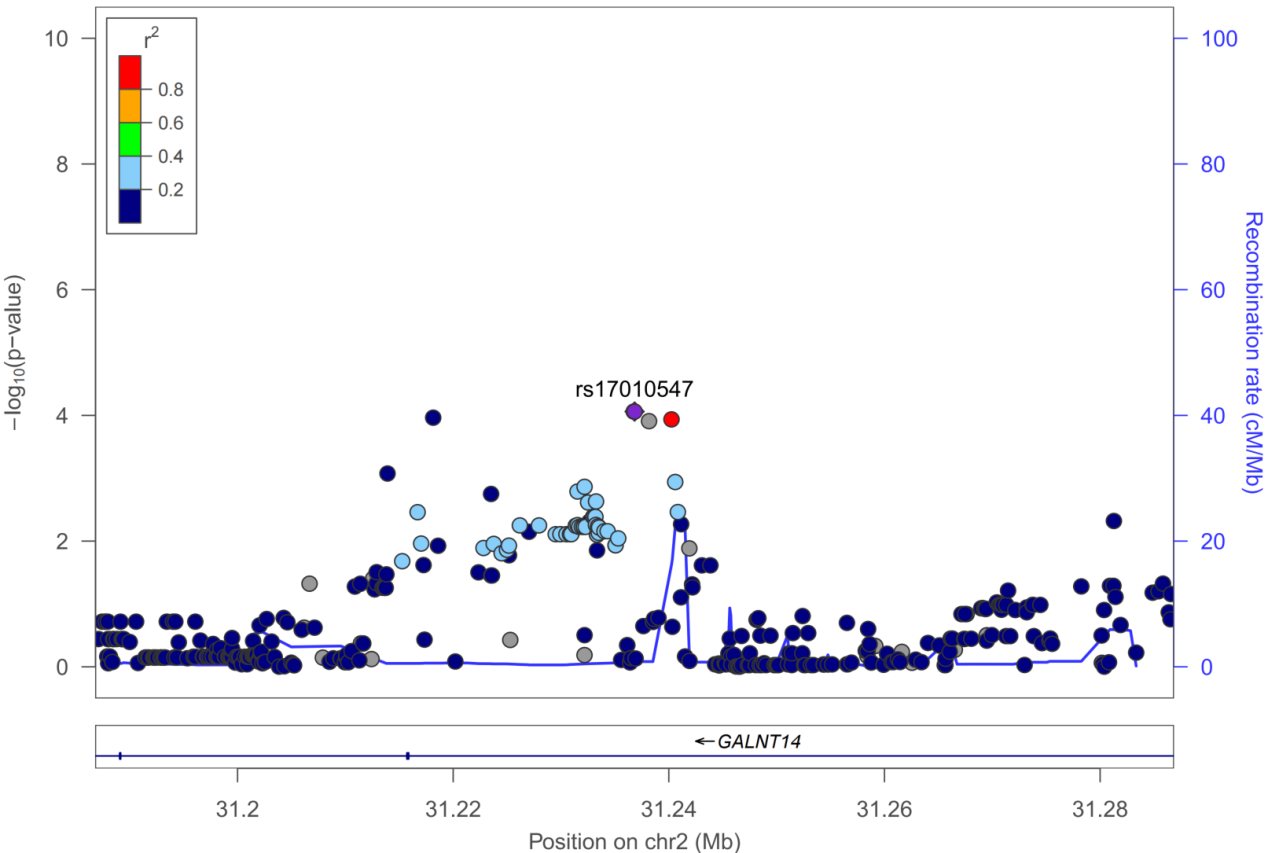 |
| **Figure S2.** Regional association plots showing ±50 kb of the gene regions in *CREB3* (A) and *GALNT14* (B). |

.

| **A** | **B** |
| --- | --- |
| 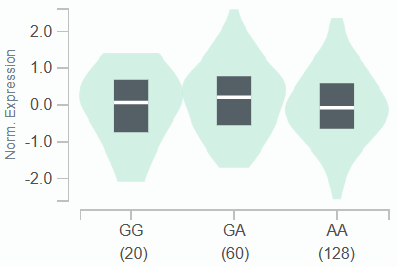 *GABARAPL1*, rs2900384, Liver  *P* = 0.090 | 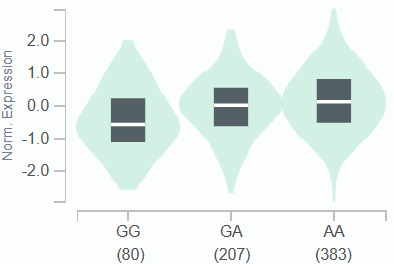 *GABARAPL1*, rs2900384, Whole Blood  *P* = 0.050 |
| **C** | **D** |
| 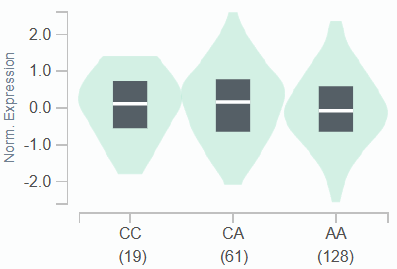 *GABARAPL1*, rs7248, Liver  *P* = 0.165 | 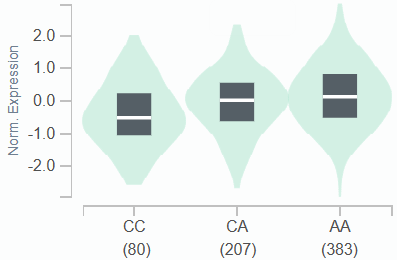 *GABARAPL1*, rs7248, Whole Blood  *P* = 0.056 |
| **E** | **F** |
| 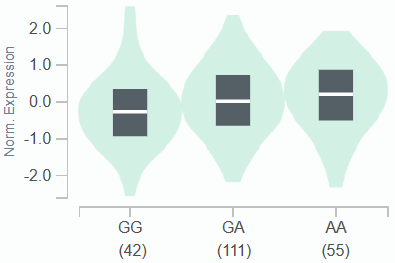 *MAP3K14*, rs708563, Liver  *P* = 0.079 | 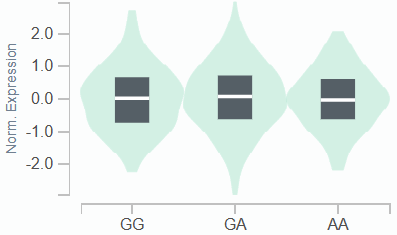 *MAP3K14*, rs708563, Whole Blood  *P* = 0.530 |
| **Figure S3**. The expression quantitative trait loci (eQTL) analysis of three SNPs. The eQTL results of *GABARAPL1* rs2900384 in normal liver tissues (A) and whole blood cells (B); The eQTL results of *GABARAPL1* rs7248 in normal liver tissues (C) and whole blood cells (D); The eQTL results of *MAP3K14* rs708563 in normal liver tissues (E) and whole blood cells (F).  No results were found for rs869199580, rs1214223169, rs1399974412, and rs8033106 in neither normal liver tissues, nor whole blood cells. | |

| **A** |
| --- |
| 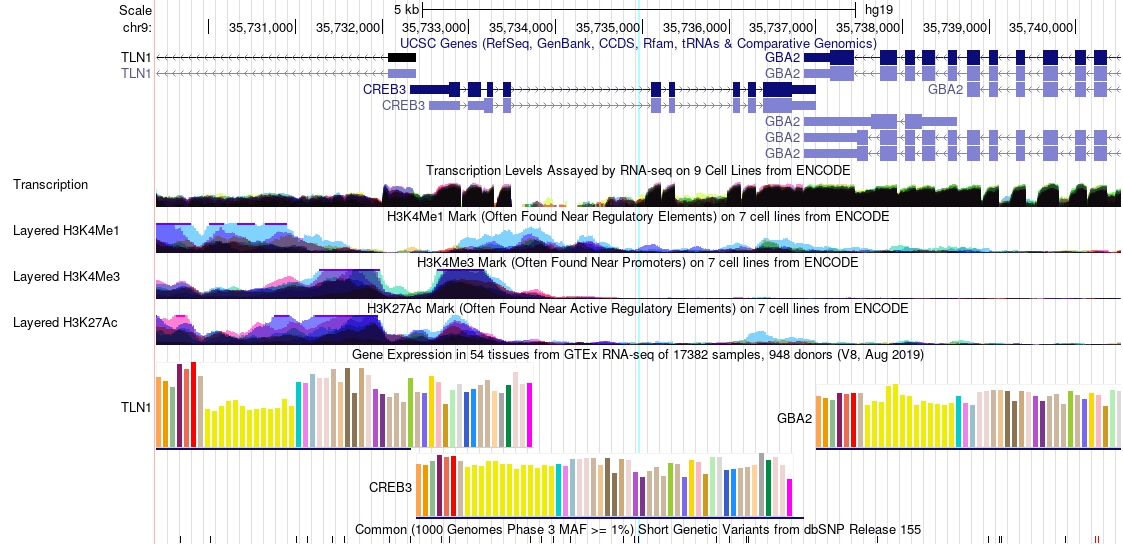 rs10814274 |
| **B** |
| 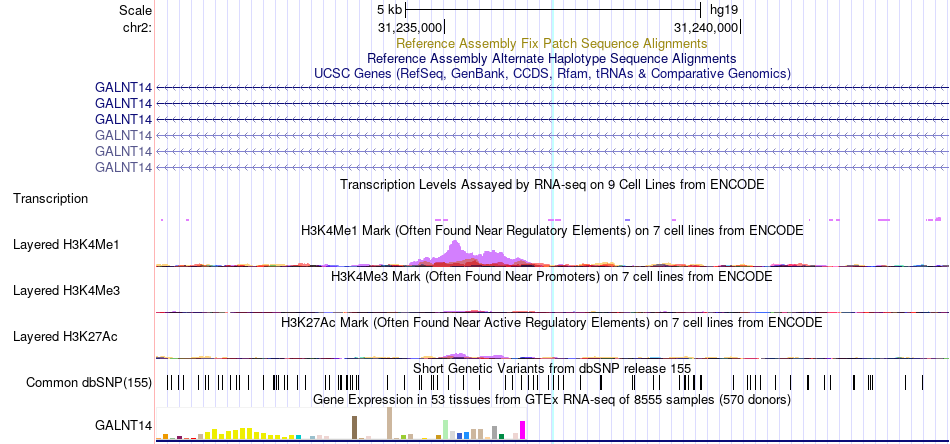 rs17010547 |
| **Figure S4.** Functional prediction of two selected SNPs in the ENCODE project. Location and functional prediction of *CREB3* rs10814274 (A) and *GALNT14* rs17010547 (B). |

.

| 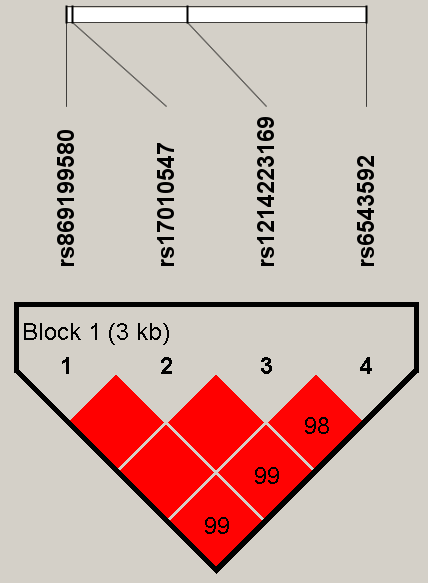 |
| --- |
| **Figure S5**. The linkage disequilibrium plot of SNPs of *GALNT14* |

| **A** | **B** |
| --- | --- |
| 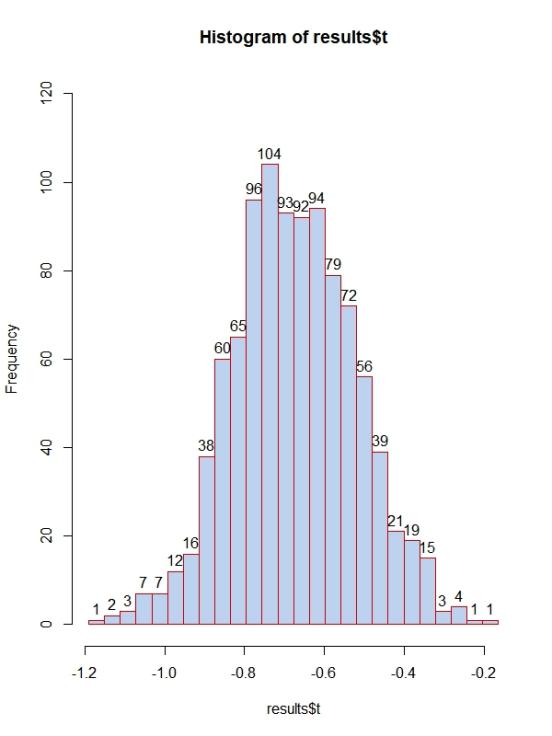 log2HR  ***P* (Shapiro.test) = 0.714**  **95% CI of log2HR (0.51-0.78)** | 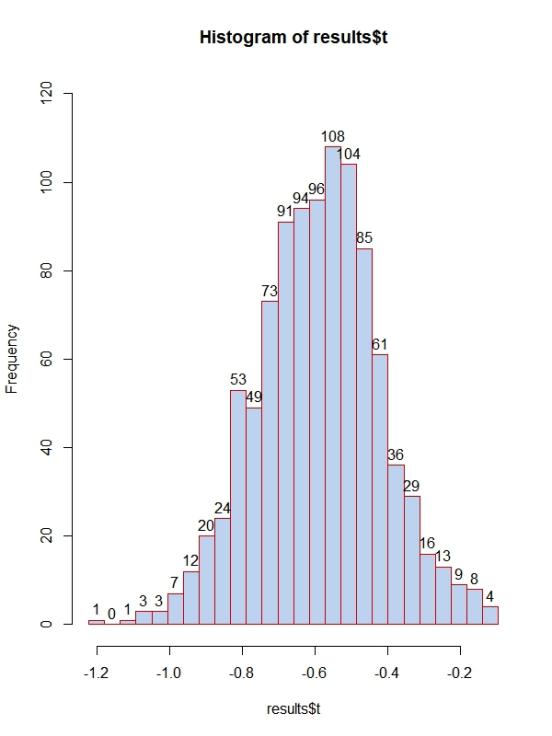 ***P* (Shapiro.test) = 0.237**  **95% CI of log2HR (0.52-0.84)**  log2HR |
| **Figure S6**. The *P* value and 95% confidence interval (CI) for the distribution histogram of 1000 hazards ratio (HR) values of *CREB3* rs10814274 (A) and *GALNT14* rs17010547 (B) after grouped by bootstrapping for 1000 times. | |

.

| **Table S1**. List of 480 ferroptosis-related genes | | |  |
| --- | --- | --- | --- |
| **Geneset** | **Selected genes^a^** | **Number of genes** | |
| Driver | *ABCC1,ACADSB,ACO1,ACSF2,ACSL1,ACSL4,ACVR1B,ADAM23,AEBP2,AGPAT3,AGPS,ALOX12,ALOX12B,ALOX15,ALOX5,ALOXE3,AMN,ANO6,AQP3,AQP5,AQP8,ARHGEF26-AS1,ASMTL-AS1,ATF3,ATF4,ATG13,ATG16L1,ATG3,ATG4D,ATG5,ATG7,ATM,ATP5MC3,BACH1,BAP1,BECN1,BID,BRD7,BRPF1,CARS1,CCDC6,CD82,CDCA3,CDKN2A,CDO1,CFL1,CGAS,CHAC1,CHP1,CIRBP,CLTRN,COX4I2,CPEB1,CS,CTSB,CYB5R1,CYBB,CYGB,CYP4F8,DCAF7,DDR2,DLD,DNAJB6,DPEP1,DPP4,DUOX1,DUOX2,EGFR,EGLN2,EGR1,ELAVL1,ELOVL5,EMC2,EPAS1,EPT1,FADS1,FADS2,FAR1,FBXW7,FLT3,FOXO4,G6PD,G6PDX,GABARAPL1,GABARAPL2,GJA1,GLS2,GOT1,GPAT4,GRIA3,GSK3B,GSTZ1,H19,HDDC3,HIF1A,HILPDA,HMGB1,HMOX1,HOTAIR,HRAS,IDH1,IDO1,IFNA1,IFNA10,IFNA13,IFNA14,IFNA16,IFNA17,IFNA2,IFNA21,IFNA4,IFNA5,IFNA6,IFNA7,IFNA8,IFNG,IL1B,IL6,INTS2,IREB2,KDM5A,KDM5C,KDM6B,KEAP1,KLF2,KMT2D,KRAS,LCE2C,LGMN,LIFR,LIG3,LONP1,LPCAT3,LPIN1,LYRM1,MAP1LC3A,MAP3K11,MAP3K14,MAPK1,MAPK14,MAPK3,MAPK9,MDM2,MDM4,MEG3,METTL14,MFN2,MIB1,MIB2,MICU1,MIOX,MLLT1,MMD,MT1DP,MTCH1,MTDH,MYB,MYCN,NCOA4,NDRG1,NOX1,NOX3,NOX4,NOX5,NR1D1,NR1D2,NRAS,OSBPL9,PANX1,PAQR3,PEBP1,PEX10,PEX12,PEX2,PEX3,PEX6,PGD,PGRMC1,PHF21A,PHKG2,PIEZO1,PIK3CA,POM121L12,POR,PPARG,PRKAA1,PRKAA2,PRKCA,PTEN,PTPN6,PVT1,QSOX1,RPL8,SAT1,SCP2,SIRT1,SLC11A2,SLC1A5,SLC25A28,SLC38A1,SLC39A14,SLC39A7,SLC7A11,SMAD7,SMG9,SMPD1,SNCA,SNX4,SNX5,SOCS1,STING1,TAFAZZIN,TBK1,TF,TFR2,TFRC,TGFB1,TGFBR1,TIMM9,TIMP1,TLR4,TNFAIP3,TOR2A,TP53,TRIM21,TRIM26,TRIM46,TSC1,TTPA,ULK1,ULK2,USP11,USP7,VDAC2,WIPI1,WIPI2,WWTR1,YAP1,YTHDC2,YY1AP1,ZEB1,ZFAS1* | 242 | |
| Marker | *CHAC1,FTH1, FTH1,GPX4,GPX4,HSPB1,NFE2L2,PTGS2,SLC40A1,TF,TFRC* | 9 | |

| **Table S1**. List of 480 ferroptosis-related genes (continued) | | |  |
| --- | --- | --- | --- |
| **Geneset** | **Selected genes^a^** | **Number of genes** | |
| Suppressor | *ABCC5,ABHD12,ACOT1,ACSL3,ADAMTS13,ADIPOQ,AHCY,AIFM2,AKR1C1,AKR1C2,AKR1C3,AKT1S1,ALDH3A2,AR,ARF6,ARNTL,ASAH2,ATF2,ATF4,BCAT2,BEX1,BRD2,BRD3,BRD4,BRDT,CA9,CAMKK2,CAV1,CBS,CD44,CDC25A,CDH1,CDKN1A,CHMP1A,CHMP5,CHMP6,CISD1,CISD2,CISD3,COPZ1,CP,CREB1,CREB3,CREB5,DAZAP1,DECR1,DHODH,ECH1,ENO3,ENPP2,ETV4,EZH2,FABP4,FADS2,FANCD2,Fer1HCH,FGF21,FH,FNDC5,FTH1,FTL,FTMT,FURIN,FXN,FZD7,G6PD,GALNT14,GCH1,GCLC,GDF15,GLRX5,GOT1,GPX4,GSTM1,HCAR1,HELLS,HIF1A,HMOX1,HSF1,HSPA5,HSPB1,IDH2,IL6,ISCU,JUN,KDM3B,KDM4A,KIF20A,KLHDC3,LAMP2,LCN2,MAPKAP1,MARCHF5,MEF2C,MEG8,MGST1,MLST8,MPC1,MS4A15,MT1G,MTF1,MTOR,MUC1,NCOA3,NEAT1,NEDD4,NEDD4L,NF2,NFE2L2,NFS1,NOS2,NQO1,NR4A1,NR5A2,NT5DC2,NUPR1,OIP5AS1,OTUB1,P4HB,PANX2,PARK7,PARP1,PARP10,PARP11,PARP12,PARP14,PARP15,PARP16,PARP2,PARP3,PARP4,PARP6,PARP8,PARP9,PDK4,PDSS2,PEDS1,PIK3CA,PIR,PLA2G6,PLIN2,PML,PPARA,PPARD,PPP1R13L,PRDX1,PRDX6,PRKAA2,PROK2,PROM2,PRR5,PTPN18,RARRES2,RB1,RBMS1,RELA,RHEBP1,RICTOR,RNF113A ,RPTOR,RRM2,SCD,SENP1,SESN2,SIAH2,SIRT1,SIRT2,SIRT3,SIRT6,SLC16A1,SLC3A2,SLC40A1,SLC7A11,SMPD1,SOX2,SQSTM1,SRC,SREBF1,SREBF2,SRSF9,STAT3,STK11,SUV39H1,TERT,TF,TFAM,TFAP2A,TFRC,TMBIM4,TMEM161B-DT,TMSB4X,TMSB4Y,TP53,TP63,TRIB2,TXN,TYRO3,USP11,USP35,VCP,VDAC2,VDR,ZFP36* | 203 | |
| Unclassified | *ACSF2,AGPAT3,ALB,ALOX12,ALOX15,ALOX5,ANGPTL7,ARRDC3,ASNS,ATF3,ATF4,ATP5MC3,ATP6V1G2,AURKA,BLOC1S5-TXNDC5,BNIP3,CAPG,CBR1,CBS,CEBPG,CXCL2,DDIT3,DDIT4,DRD4,DRD5,DUSP1,EIF2AK4,EIF2S1,ELAVL1,FTH1,FTL,GABPB1,GDF15,GLUT13,GPT2,GPX2,GPX4,HAMP,HBA1,HERPUD1,HIC1,HMGB1,HMOX1,HNF4A,HSD17B11,IL33,IL6,IREB2,JDP2,KIM-1,KLHL24,LRRFIP1,LURAP1L,MAFG,MAP3K5,MAPK14,MMP13,MT3,NCF2,NFE2L2,NGB,NNMT,NOS2,OXSR1,PCK2,PLIN4,PRDX1,PSAT1,RELA,RGS4,RIPK1,RPL8,RRM2,SELENOS,SESN2,SETD1B,SLC1A4,SLC2A1,SLC2A12,SLC2A14,SLC2A3,SLC2A6,SLC2A8,SLC3A2,SLC7A11,SLC7A5,SNORA16A,SP1,SRXN1,STEAP3,STMN1,TF,TFAP2C,TFRC,TRIB3,TSC22D3,TUBE1,TXNIP,TXNRD1,UBC,VEGFA,VLDLR,XBP1,YWHAE,ZFP69B,ZNF419* | 106 | |
| **Total** |  | **480** | |
| ^a^Circular RNA, long non-coding RNA and microRNA had been removed. | | |  |
| ^b^Fer1HCH, the dupicated genes and genes in sex chromosome had been removed. | | |  |
| Website: http://www.zhounan.org/ferrdb/current/ | | |  |

| **Table S2.** Associations of demographics and clinical characteristics with survival of HBV-HCC patients. | | | | | | | |
| --- | --- | --- | --- | --- | --- | --- | --- |
| **Parameter** | **Combined dataset（n=866）** | | **MST** | **Univariate analysis** | | **Multivariable analysis** | |
|  | **All** | **Death (%)** | **(Month)** | **HR**  **(95% CI)** | ***P*** | **HR**  **(95% CI)**^a^ | ***P***^a^ |
| Age |  |  |  |  |  |  |  |
| ≤ 47 | 434 | 233 (53.69) | 47 | 1.00 |  | 1.00 |  |
| > 47 | 432 | 186 (43.06) | 82.6 | 0.72 (0.59-0.87) | **<0.001** | 0.81 (0.66-0.99) | **0.036** |
| Sex |  |  |  |  |  |  |  |
| Female | 106 | 42 (39.62) | 66.97 | 1.00 |  | 1.00 |  |
| Male | 760 | 377 (49.61) | 71.98 | 1.25 (0.90-1.71) | 0.179 | 1.26 (0.90-1.76) | 0.176 |
| Smoking |  |  |  |  |  |  |  |
| No | 545 | 268 (49.17) | 62 | 1.00 |  | 1.00 |  |
| Yes | 321 | 151 (47.04) | 60 | 0.96 (0.79-1.18) | 0.724 | 0.91 (0.71-1.17) | 0.475 |
| Drinking |  |  |  |  |  |  |  |
| No | 614 | 292 (47.56) | 66 | 1.00 |  | 1.00 |  |
| Yes | 252 | 127 (59.40) | 54 | 1.07 (0.87-1.32) | 0.5 | 1.08 (0.84-1.41) | 0.541 |
| AFP (ng/ml) |  |  |  |  |  |  |  |
| ≤400 | 522 | 232 (44.44) | 70.3 | 1.00 |  | 1.00 |  |
| >400 | 344 | 187 (54.36) | 39.1 | 1.59 (1.31-1.93) | **<0.001** | 1.29 (1.05-1.57) | **0.015** |
| Cirrhosis |  |  |  |  |  |  |  |
| No | 390 | 184 (47.18) | 61.9 | 1.00 |  | 1.00 |  |
| Yes | 476 | 235 (49.37) | 61.5 | 1.00 (0.82-1.22) | 0.996 | 1.04 (0.85-1.41) | 0.702 |
| Embolus |  |  |  |  |  |  |  |
| No | 636 | 260 (40.88) | 90.6 | 1.00 |  | 1.00 |  |
| Yes | 230 | 159 (69.13) | 23.1 | 2.73 (2.32-3.34) | **<0.001** | 1.74 (1.38-2.21) | **<0.001** |
| BCLC stage |  |  |  |  |  |  |  |
| 0/A | 427 | 146 (34.19) | 99.6 | 1.00 |  | 1.00 |  |
| B/C | 439 | 273 (62.19) | 30.1 | 2.73 (2.23-3.35) | **<0.001** | 1.98 (1.56-2.52) | **<0.001** |
| Abbreviation: HBV-HCC, hepatitis B virus-related hepatocellular carcinoma; MST, median survival time; HR, hazards ratio; AFP, serum alpha-fetoprotein; BCLC, the Barcelona Clinic Liver Cancer. | | | | | | | |
| ^a^ Multivariate Cox regression analyses were adjusted for age, sex, smoking, drinking, AFP, cirrhosis, embolus and BCLC stage. | | | | | | | |
| The results were in **bold**, if P<0.05. | | | | | | | |

| **Table S3.** Functional prediction of three identified ferroptosis-related SNPs and SNPs in high linkage disequilibrium | | | | | | | | | | |
| --- | --- | --- | --- | --- | --- | --- | --- | --- | --- | --- |
| **SNP** | **LD (*r^2^*)** | **Chr** | **Position** | **Allele** | **SNPinfo^a^** | **RegulomeDB^b^** | **HaploReg V4.2c** | | | |
|  |  |  |  |  | **TFBS** | **Rank** | **Enhancer**  **histone marks** | **Motifs change** | **GENCODE gene** | **dbSNP func annot** |
| **rs10814274** | 1 | 9 | 35734956 | C>T | Y | 6 |  | E2A,TAL1 | *CREB3* | intronic |
| rs1570249 | 0.99 | 9 | 35752255 | A>G | Y | 4 | VAS | - | *RGP1* | intronic |
| rs3750434 | 0.97 | 9 | 35739879 | A>G | - | 5 | SKIN | NRSF | *GBA2* | intronic |
| rs148542284 | 0.94 | 9 | 35731419 | C>CCT | - | - |  | Cdc5,NF-kappaB | *TLN1* | intronic |
| rs201970157 | 0.92 | 9 | 35731418 | T>TC | - | 4 |  | Cdc5,Gfi1,NF-kappaB | *TLN1* | intronic |
| rs1570246 | 0.9 | 9 | 35748809 | T>G | Y | 4 |  | Egr-1,SRF | *GBA2* | 5'-UTR |
| rs7862695 | 0.9 | 9 | 35766119 | T>C | - | 5 | BLD | AP-1 | *6kb 5' of RP11-112J3.16* |  |
| rs10758322 | 0.88 | 9 | 35776193 | T>C | - | 2b |  | 11 altered motifs | *RP11-112J3.16* |  |
| rs10758321 | 0.87 | 9 | 35767756 | A>G | - | 1f |  |  | *4.4kb 5' of RP11-112J3.16* |  |
| rs2381400 | 0.87 | 9 | 35776422 | A>G | - | 4 | 6 tissues | STAT | *RP11-112J3.16* |  |

| **Table S3**. Functional prediction of three identified ferroptosis-related SNPs and SNPs in high linkage disequilibrium (continued) | | | | | | | | | | |
| --- | --- | --- | --- | --- | --- | --- | --- | --- | --- | --- |
| **SNP** | **LD (*r^2^*)** | **Chr** | **Position** | **Allele** | **SNPinfo^a^** | **RegulomeDB^b^** | **HaploReg V4.2^c^** | | | |
|  |  |  |  |  | **TFBS** | **Rank** | **Enhancer histone marks** | **Motifs change** | **GENCODE gene** | **dbSNP func annot** |
| **rs17010547** | 1 | 2 | 31013958 | T>C | - | 7 | BLD | FXR | *GALNT14* | intronic |
| rs58520251 | 1 | 2 | 31013882 | T>TG | - | 5 |  | 4 altered motifs | *GALNT14* | intronic |
| **rs6543592** | 0.94 | 2 | 31017380 | A>G | - | 7 |  | GR,NR4A | *GALNT14* | intronic |
| ^a^ RegulomeDB: http://regulomedb.org； | | | | | | | | | | |
| ^b^ SNPinfo: https://snpinfo.niehs.nih.gov/snpinfo/selegwas.html; | | | | | | | | | | |
| ^c^ HaploReg: https://pubs.broadinstitute.org/mammals/haploreg/haploreg.php  Abbreviations: SNP, single nucleotide polymorphism; LD, linkage disequilibrium; TFBS, transcription factor binding site. | | | | | | | | | | |

| **Table S4.** Stepwise multivariate Cox regression analysis for the identification of significant SNPs in ferroptosis-related genes in HBV-HCC patients. | | | | |
| --- | --- | --- | --- | --- |
| **Variables** | **Category** | **Frequency** | **HR (95% CI)^a^** | ***P*^a^** |
| Age | ≤47/>47 | 434/432 | 0.76 (0.63-0.93) | **0.007** |
| AFP (ng/mL) | ≤400/>400 | 522/344 | 1.33 (1.09-1.63) | **0.005** |
| Embolus | NO/YES | 636/230 | 1.78 (1.41-2.26) | **<0.001** |
| BCLC | 0-A/B-C | 439/427 | 2.00 (1.58-2.54) | **<0.001** |
| *CREB3* rs10814274 | CC/CT/TT | 261/425/180 | 0.76 (0.66-0.88) | **<0.001** |
| *GALNT14* rs17010547 | TT/TC/CC | 592/244/30 | 0.69 (0.57-0.83) | **<0.001** |
| Abbreviation: SNP, single nucleotide polymorphisms; HBV-HCC, hepatitis B virus-related hepatocellular carcinoma; HR, hazards ratio; CI, confidence interval; AFP, serum alpha-fetoprotein; BCLC, the Barcelona Clinic Liver Cancer. | | | | |
| ^a^Obtained in a stepwise multivariate cox regression analysis, and variables included age, sex, smoking status, drinking status, cirrhosis, AFP, embolus, BCLC stage, and two SNPs identified (*CREB3* rs10814274, and *GALNT14* rs17010547). | | | | |
| The results were in **bold**, if P<0.05. | | | | |

| **Table S5**. Associations between rs9679162 and HBV-HCC survival in discovery, validation and combined dataset. | | | | | | | | | | | |
| --- | --- | --- | --- | --- | --- | --- | --- | --- | --- | --- | --- |
| SNP | Gene | MAF | Discovery dataset (n=433) | | | Validation dataset (n=433) | | | Combined dataset (n=866) | | |
|  |  |  | HR^a^ | *P^a^* | FPRP | HR^a^ | *P^a^* | FPRP | HR^a^ | *P^a^* | FPRP |
| rs9679162 | *GALNT14* | 0.470 | 0.97 (0.80-1.18) | 0.771 | 0.872 | 0.95 (0.78-1.15) | 0.605 | 0.843 | 0.96 (0.84-1.11) | 0.609 | 0.840 |
| Abbreviation: SNP, single nucleotide polymorphisms; HBV-HCC, hepatitis B virus-related hepatocellular carcinoma; MAF, minor allele frequency; FPRP, false-positive report probability; HR, Hazard Ratio. | | | | | | | | | | | |
| ^a^Adjusted for age, sex, smoking status, drinking status, AFP, embolus and BCLC. | | | | | | | | | | | |
| The results were in bold, if *P*<0.05 or FPRP<0.2. | | | | | | | | | | | |
